# Isotope Effects on the Electronic Spectra of Ammonia from Ab Initio Semiclassical Dynamics

**DOI:** 10.1021/acs.jpca.3c04607

**Published:** 2023-09-22

**Authors:** Ēriks Klētnieks, Yannick Calvino Alonso, Jiří J. L. Vaníček

**Affiliations:** Laboratory of Theoretical Physical Chemistry, Institut des Sciences et Ingénierie Chimiques, Ecole Polytechnique Fédérale de Lausanne (EPFL), Lausanne CH-1015, Switzerland

## Abstract

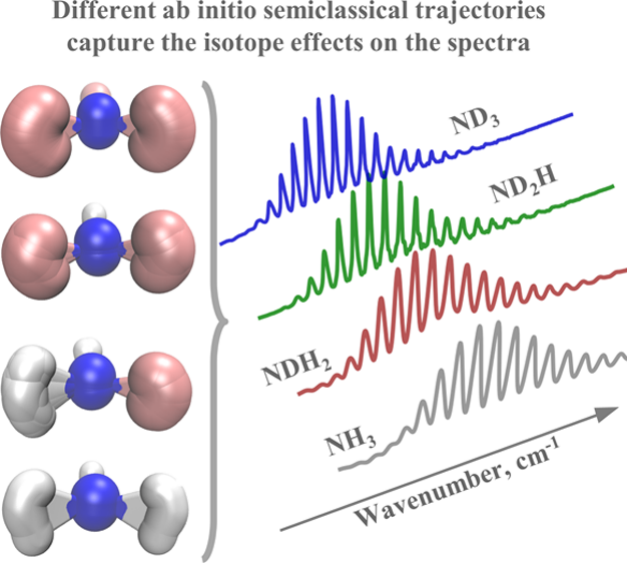

Despite its simplicity, the single-trajectory thawed Gaussian approximation has proven useful for calculating the vibrationally resolved electronic spectra of molecules with weakly anharmonic potential energy surfaces. Here, we show that the thawed Gaussian approximation can capture surprisingly well even more subtle observables, such as the isotope effects in the absorption spectra, and we demonstrate it on the four isotopologues of ammonia (NH_3_, NDH_2_, ND_2_H, and ND_3_). The differences in their computed spectra are due to the differences in the semiclassical trajectories followed by the four isotopologues, and the isotope effects—narrowing of the transition band and reduction of the peak spacing—are accurately described by this semiclassical method. In contrast, the adiabatic harmonic model shows a double progression instead of the single progression seen in the experimental spectra. The vertical harmonic model correctly shows only a single progression but fails to describe the anharmonic peak spacing. Analysis of the normal-mode activation upon excitation provides insight into the elusiveness of the symmetric stretching progression in the spectra.

## Introduction

Vibrationally resolved electronic spectroscopy has made significant contributions to the understanding of the structure and dynamics of molecules. However, extracting information about the potential energy surface (PES) on which the dynamics occur remains challenging due to several factors influencing the experimentally observed spectra. Since the electronic structure is invariant to the isotope substitution, measuring the isotope effects in the spectra not only provides additional information about the shape of the surface and dynamics but also aids in the assignment of transitions. Computational methods help to interpret experimental results and provide a clearer understanding of the dynamics responsible for the observed spectra.

Within the Born–Oppenheimer approximation,^1,2^ the isotope effects in the spectra are attributed to the changes in the characteristic frequencies of isotopologues, which depend strongly on the mass of the substituted atom. Depending on the system of interest, the isotope exchange can shift the peak positions to higher or lower energies. In general, the isotope substitution changes the energy of the 0–0 transition, the width of the transition band, and also the spacing, widths, and intensities of peaks. The magnitude of these changes depends on the mass ratio of the substituted species and on the displacement in the vibrational normal modes responsible for the spectra.^3,4^

Among the various methods for calculating the isotope effects in the spectra, the simplest approach is based on the global harmonic approximation to the potential.^5−10^ Harmonic models are computationally cheap and can capture some isotope effects correctly, but they fail in systems with large-amplitude nuclear motion.^11^ In contrast, the exact quantum dynamics on a grid^12^ or multiconfigurational time-dependent Hartree method^13^ reproduce all of the isotope effects seen in the experimental spectra, but at the high cost of constructing a global PES.^14^ A compromise is offered by the semiclassical trajectory-based methods,^15−25^ which, in terms of accuracy, lie between the harmonic and exact results. While the most accurate semiclassical methods^15,19,23−28^ can capture quantum effects accurately, achieving high accuracy often demands the propagation of a large ensemble of trajectories, which may not be computationally feasible in ab initio applications.

The single-trajectory thawed Gaussian approximation (TGA), introduced by Heller,^29^ is a semiclassical method, which is accurate for short propagation times and has been used to calculate both absorption and emission spectra of weakly anharmonic molecules with a large number of degrees of freedom.^16,30−33^ In weakly anharmonic systems and for short times relevant in electronic spectroscopy, the single Gaussian wave packet used in the TGA is, sometimes surprisingly, sufficient to sample the dynamically important region of the phase space. Moreover, the classical trajectory associated with the wavefunction provides a simplified, intuitive picture of the dynamics, while the evolving width of the Gaussian wave packet partially captures the quantum effects. Successful past applications of the TGA prompted us to apply it to the isotope effects. Although the molecular PES is approximately invariant under isotope substitution, for each isotopologue, the guiding trajectory explores a different region of this common PES. This requires propagating a new trajectory for each isotopologue, but the total computational cost is still considerably lower than the cost of constructing the full surface.

To investigate the isotope effect on the spectrum, a comprehensive understanding of the PES associated with the transition is essential. In the case of ammonia, the first excited-state  is quasi-bound in the Franck–Condon region.^34,35^ A finite barrier separates the bound region from the conical intersection of the  and  states. This conical intersection couples the two surfaces nonadiabatically and is responsible for an internal conversion, which leads to the broadening of the absorption spectra.^36−38^ The lifetime in the quasi-bound region depends on the isotopologue and ranges from a few hundred femtoseconds to a few picoseconds, allowing for more than several oscillations to occur before the escape. The ammonia absorption spectrum  (*S*_1_ ← *S*_0_) has a long progression that is induced by the activation of the symmetric bending (umbrella motion) and symmetric stretching modes.^39−41^ Although the experimental spectra of isotopologues (NDH_2_, ND_2_H, and ND_3_) have similar patterns as the spectrum of NH_3_, the isotope effects are clearly visible.^42^ As the number of hydrogen atoms substituted by deuterium increases, the energy of the 0–0 transition increases, while the peak spacing and width decrease. In addition, the transition band becomes narrower.

The isotope substitution can also affect the molecular symmetry. Although the ground-state geometry of all four ammonia isotopologues has a pyramidal shape, the NH_3_ and ND_3_ belong to the *C*_3*v*_ point group, whereas NDH_2_ and ND_2_H belong to the *C*_*s*_ point group. As the symmetry changes from one isotopologue to another, the dynamics on the excited-state surface change not only due to the change of reduced masses but also due to the activation of other normal modes.^4^

## Theory

**Figure 1:**
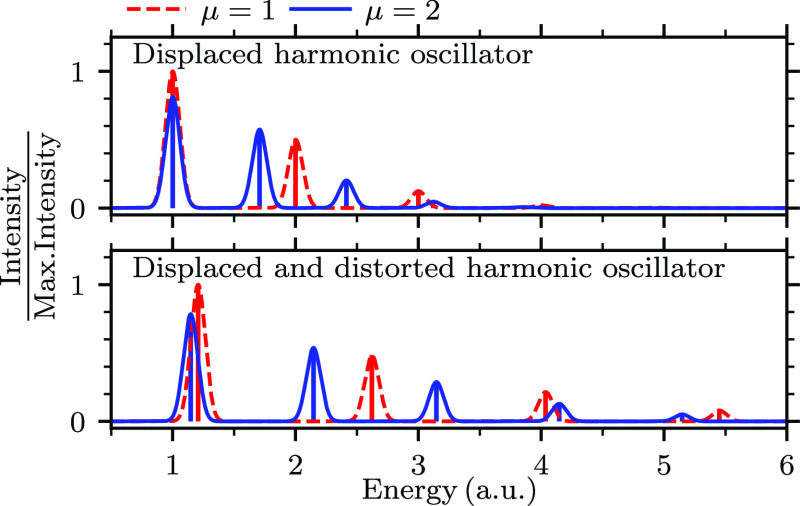
Isotope effects in the electronic spectra of one-dimensional harmonic oscillators. The finite-width peaks were obtained with the TGA, whereas the vertical lines indicate the spectra obtained with the time-independent approach,^44,45^ in which the Franck–Condon factors were calculated with eq 1 (in the top panel) or eq 3.26 from ref (43) (in the bottom panel). The spectra were scaled according to the maximum intensity of the system with μ = 1. Details of this calculation can be found in Section A of the Supporting Information.

To demonstrate various isotope effects on electronic spectra, let us first consider an analytically solvable one-dimensional displaced harmonic oscillator model. In this model, the two PESs involved in the transition are described by harmonic oscillators with the same force constant (*k*_g_ = *k*_e_ = *k*), but whose minima are displaced horizontally by Δ*q* and vertically by an energy gap Δ*E*. Assuming that the initial state is the vibrational ground state |Ψ_g,0_⟩ of the electronic ground state, the Franck–Condon factors, which determine the transition probability between the two vibronic states and hence the intensities of the spectral peaks, follow the Poisson distribution
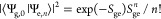
1where  is the Huang-Rhys parameter for the ground and excited states, , and μ is the reduced mass of the oscillator. Analytical expressions for Franck–Condon factors for squeezed and displaced harmonic oscillators (in which *k*_e_ ≠ *k*_g_) can be found, e.g., in ref (43). The spectra computed in these model systems with the time-independent approach are compared with the results obtained with the TGA, which is exact in quadratic potentials, in Figure 1. In spite of the simplicity of the models, the spectra exhibit all of the abovementioned isotope effects—namely, the changes in the 0–0 transition, peak spacing, and width of the transition band—except for the effect on the peak widths, which is zero in the time-independent approach and arbitrary in the TGA (due to the choice of the damping of the wave packet time autocorrelation function). Note that the 0–0 transition is only affected in the distorted model, where the vibrational frequencies of the ground and excited states are different.

Polyatomic molecules with at least two vibrational degrees of freedom pose an additional challenge due to the multidimensional nature of their PESs. Within the harmonic approximation, the excited-state PES *V*(*q*) of a molecule is approximated by a quadratic expansion about the reference geometry *q*_ref_
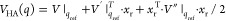
2where *x*_r_ ≔ *q* – *q*_ref_,  is the gradient vector, and  is the Hessian matrix at the reference geometry. The PES of the excited state is generally not only displaced and distorted but also rotated with respect to the surface of the initial state. This rotation is called the Duschinsky effect and is characterized by the Duschinsky matrix *J* that relates the normal-mode coordinates of the two states.^46^ The general form of the potential in eq 2 allows for a free choice of *q*_ref_, but two choices are the most natural. The adiabatic harmonic (AH) model is constructed about the equilibrium geometry of the excited-state surface, whereas the vertical harmonic (VH) model expands the excited-state surface about the Franck–Condon geometry, i.e., the equilibrium geometry of the ground-state surface.^6,47^ Various exact and efficient algorithms are able to treat global harmonic models of large systems, and the thawed Gaussian approximation is one of them. The harmonic models are easy to construct as they only require a single Hessian calculation in the ground and excited states once the geometries have been optimized. The subsequent evaluation of the autocorrelation functions and spectra requires much less computational effort. The drawback of the harmonic models is the complete neglect of anharmonicity, which may lead to erroneous results for systems with large-amplitude nuclear motion.

Going beyond the harmonic approximation requires the inclusion of anharmonicity effects, and Heller’s^29^ thawed Gaussian approximation does it at least partially by employing the local harmonic approximation
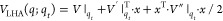
3of the potential about the current center *q*_*t*_ of the wave packet (*x* ≔ *q* – *q*_*t*_). The TGA allows the exploration of anharmonic parts of the potential without the need to construct a full PES a priori. Within the time-dependent approach to spectroscopy,^44,48^ the nuclear wave packet is propagated by solving the time-dependent Schrödinger equation
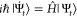
4where *Ĥ* ≔ *p̂*^T^·*m*^−1^·*p̂*/2 + *V*(*q̂*) is the vibrational Hamiltonian, |Ψ_*t*_⟩ is the nuclear wave packet, and *m* = diag(*m*_1_, ..., *m*_D_) is the diagonal mass matrix. The thawed Gaussian approximation assumes that the nuclear wave packet is a *D*-dimensional Gaussian,^49,50^ which, in Hagedorn’s parameterization,^51,52^ is written as
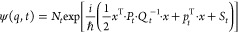
5where  is the normalization constant, *x* ≔ *q* – *q*_*t*_ is the shifted position, *q*_*t*_ and *p*_*t*_ are the position and momentum of the wave packet’s center, *S*_*t*_ is the classical action, and *P*_*t*_ and *Q*_*t*_ are complex *D* × *D* matrices, which satisfy the relations
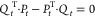
6
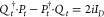
7where *I*_D_ is the *D* × *D* identity matrix. Approximating *V* with *V*_LHA_ in the Schrödinger eq 4 yields the nonlinear Schrödinger equation
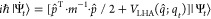
8This equation is solved exactly by the Gaussian ansatz if the Gaussian parameters satisfy the first-order differential equations
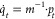
9
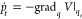
10
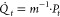
11
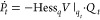
12
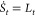
13where *L*_*t*_ denotes the Lagrangian
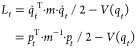
14Equations 9 and 10 imply that the center of the wave packet follows exactly the classical trajectory of the original potential, while the width of the Gaussian is propagated with the effective potential  [see eqs 11 and 12].

Being a single-trajectory method, the TGA can be easily combined with an on-the-fly evaluation of the electronic structure. Most often, electronic structure calculations for a molecule with *N* atoms are performed in 3*N* Cartesian coordinates. However, natural coordinates for the propagation of the thawed Gaussian wave packet and for the construction of global harmonic models are the 3*N* – 6 mass-scaled vibrational normal-mode coordinates *q*_*j*_. We perform the propagation in the excited-state vibrational normal-mode coordinates since these coordinates provide the most natural description of the dynamics following the electronic excitation. While it is possible to employ a different set of normal-mode coordinates, utilizing other modes would complicate the discussion of the normal-mode evolution. Nevertheless, we have confirmed that employing the ground-state normal-mode coordinates, which minimize the initial rovibrational coupling, had a negligible impact on the computed spectra of ammonia isotopologues (not shown). Because the question of the optimal choice of the reference geometry and coordinates is nontrivial and system-dependent, we plan to address it in more detail in future work.^53^

The transformation from Cartesian to normal-mode coordinates requires the removal of translational and rotational degrees of freedom. Although the vibrations and rotations are not fully separable, we reduce the coupling by translating and rotating the nuclei of the molecule into the Eckart frame. Here, we closely follow the procedure described in more detail in ref (48).

Let us denote the full molecular configuration as ξ ≔ (**r**_1_, ..., **r**_*N*_), where **r**_*a*_ (*a* = 1, ..., *N*) are the Cartesian coordinates of atom *a*. We introduce a reference molecular configuration ξ_ref_ ≔ (**r**_ref,1_, ..., **r**_ref,*N*_). In all our calculations, ξ_ref_ is the equilibrium geometry on the PES of the excited state  of ammonia. First, the translational degrees of freedom are removed by shifting the coordinates of each atom to the center-of-mass frame
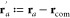
15where **r**_com_ = (∑_*a*=1_^*N*^*m*_*a*_**r**_*a*_)/(∑_*a*=1_^*N*^*m*_*a*_) and *m*_*a*_ are the masses of the nuclei of atom *a*. The translated molecular configuration is ξ^′^ ≔ (**r**_1_^′^, ..., **r**_*N*_^′^). In the following, we assume that the center of mass of the reference configuration ξ_ref_ is at the origin, i.e., **r**_ref,com_ = 0.

In the second step, we minimize the rovibrational coupling by rotating the configuration ξ′ into the Eckart frame. This is equivalent to minimizing the squared mass-scaled distance^54^
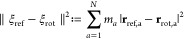
16between the rotated configuration ξ_rot_ and the reference configuration ξ_ref_. Here **r**_rot,a_ ≔ *R*·**r**_*a*_^′^. The required 3 × 3 rotation matrix *R* can be found, e.g., by the Kabsch algorithm.^55^ The transformation to and from the normal-mode coordinates is based on the orthogonal matrix *O*_ref_ that diagonalizes the mass-scaled Cartesian Hessian matrix at ξ_ref_ on the excited-state surface *V*
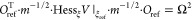
17where *m* and Ω are 3*N* × 3*N* diagonal matrices containing, respectively, the values of the atomic masses and normal-mode frequencies (still including the zero frequencies). After projecting out the zero-frequency modes associated with rotations and translations, the overall transformation from the Cartesian coordinates ξ to the mass-scaled vibrational normal-mode coordinates *q* is
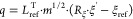
18where *L*_ref_ is the leading 3*N* × (3*N* – 6) submatrix of *O*_ref_ and R_ξ_ = *I*_*N*_ ⊗ *R* is a 3*N* × 3*N* block-diagonal matrix, whose *N* 3 × 3 blocks are identical and equal to the rotation matrix *R*. Similarly, we can obtain the potential gradient and Hessian in vibrational normal-mode coordinates
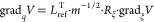
19
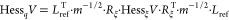
20Equation 18 can be rearranged to transform the normal-mode coordinates back to the Cartesian coordinates as
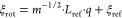
21The above framework allows combining ab initio calculations in Cartesian coordinates with the propagation of the thawed Gaussian wave packet in normal-mode coordinates. Equations 18–20 are also used for the construction of global harmonic models.

**Figure 2:**
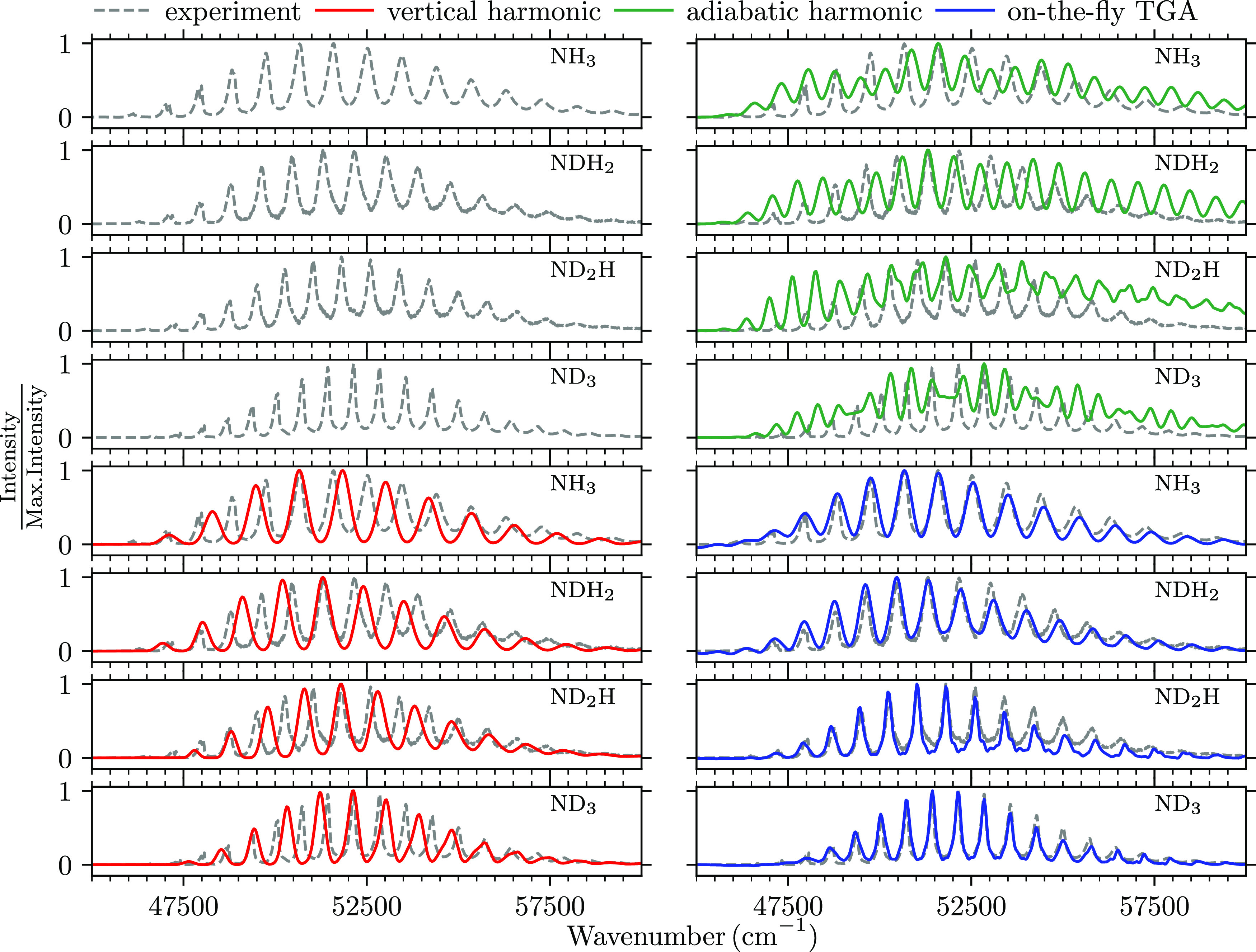
Simulated electronic absorption spectra of ammonia isotopologues (NH_3_, NDH_2_, ND_2_H, and ND_3_) are compared to the experimental spectra^42^ recorded in the gas phase at 298 K.

In what follows, all ab initio calculations of ammonia were performed using the complete active-space second-order perturbation theory CASPT2(8/8) method with Dunnings correlation-consistent basis set *aug-cc-pVTZ*; a level shift of 0.2 a.u. was applied to avoid the intruder-state problem.^56,57^ All of the calculations were performed with the Molpro2019 package.^58^ The Gaussian wave packet was propagated with a time step of 8 a.u. for 1000 steps (1 a.u. ≈ 0.024 fs, resulting in a total time of 8000 a.u. ≈ 193.5 fs) using the second-order symplectic integrator.^59^ To remove the systematic errors of the ab initio vertical excitation energies, all computed spectra in Figure 2 were shifted independently to obtain the best fit to the experiment. In addition, the spectra were broadened by a Lorentzian with a half-width at half-maximum of 170, 137, 95, and 110 cm^–1^ for NH_3_, NDH_2_, ND_2_H, and ND_3_, respectively. Neither the overall shift nor the broadening affects the subsequent analysis of peak spacing, peak intensities, and width of the spectral envelope, which are independent of the shift and broadening. Additional details can be found in Section C of the Supporting Information.

The initial Gaussian wave packet was the ground vibrational eigenstate of the harmonic fit to the PES of the ground electronic state () at one of the two degenerate minima. The initial position *q*_0_ in normal-mode coordinates was obtained from eq 18, where ξ′ is replaced with the Cartesian coordinates ξ_init_ corresponding to the equilibrium geometry of the ground state. The initial momentum *p*_0_ of the wave packet was zero. The initial *Q*_0_ and *P*_0_ matrices, which control the width of the Gaussian wave packet, were
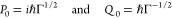
22where  is the Hessian in normal-mode coordinates calculated at the equilibrium geometry of the ground state. One could potentially include some anharmonicity of the ground state by using an optimal Gaussian initial wave packet other than the ground vibrational state of the harmonic fit, but this would be inconsistent with the local harmonic approximation employed during the excited-state propagation.

## Results and Discussion

The experimental spectra of ammonia isotopologues, shown in the top left-hand panel of Figure 2, clearly exhibit the isotope effects on the 0–0 transition energy, on the width of the transition band, and on the peak spacing, width, and intensity. First, the shift in the 0–0 transition toward higher energies can be directly compared with the adiabatic excitation energy from ab initio calculations in Table 1, indicating that quantum chemical calculations correctly reproduce the trend. Second, the bands associated with the  transition become narrower. Third, the decrease in peak spacing for highly substituted species is in line with the fact that the spacing between the vibrational energy levels decreases with increasing reduced mass. Finally, the vibronic peak widths decrease from NH_3_ to ND_3_.^60,61^ The peak widths are influenced by several factors, including the rotational contour and nonradiative processes such as tunneling and internal conversion, which lead to predissociation. The tunneling through the predissociation barrier can explain the drastic decrease in peak widths for heavier isotopologues, which have a longer lifetime in the quasi-bound region due to slower tunneling.^38,60,61^ In our calculations, which include neither rotations nor tunneling, we cannot quantify the contributions of the rotational contour and predissociation to the peak width. Therefore, we must incorporate the peak width phenomenologically.

**Table 1:** Isotope Effect on the 0–0 Transition of Ammonia Evaluated as the Difference Δ*E*_00_(ND_*j*_H_*i*_) – Δ*E*_00_(NH_3_)

	NDH_2_	ND_2_H	ND_3_
experiment	171	347	532
adiabatic excitation energy^a^	128	260	399

a The adiabatic excitation energy includes the zero-point vibrational energy. All values shown are in cm^–1^.

The AH model is constructed around the excited-state equilibrium geometry and the same Cartesian Hessian is used to construct the PES for all isotopologues. The calculated spectra in the top right-hand panel of Figure 2 show a double progression. In addition, for all isotopologues, the spectral envelope extends to higher energies, reflecting the poor description of the short-time dynamics of the system. Overall, this confirms that the AH model is a bad approximation of the strongly anharmonic PES of the ammonia molecule, where the differences between ground- and excited-state equilibrium geometries are significant. In the following, we do not discuss the isotope effect on the 0–0 transition, since the shift applied to compensate for the overall errors of the electronic structure calculations (Table S6 in the Supporting Information) is of the same order as the expected isotope effect. Instead, we focus on the isotope effects that are independent of this shift.

As observed in ref (31), the VH model in the case of NH_3_ yields a single progression and recovers the overall shape of the experimental spectrum. The results for other isotopologues further confirm this observation. Moreover, the computed envelopes of the spectra of all isotopologues agree rather well with the envelopes of the experimental spectra. This is because the VH model approximates the PES well in the Franck–Condon region, which determines the spectral envelope. The widths of spectral envelopes are predicted rather accurately (see Table 2). The model also describes qualitatively the trend of decreasing peak spacing. However, as the model fails to capture anharmonicity, the peak spacing is not reproduced quantitatively.

**Table 2:** Width^a^ of the Spectral Envelope in cm^–1^

	NH_3_	NDH_2_	ND_2_H	ND_3_
experiment	5554	5030	4700	4638
TGA	5426	5342	4792	4558
VH model	5466	5202	4808	4490
AH model	7044	7224	5540	5748

a The width of the spectral envelope was estimated as twice the square root of the weighted variance of the peak positions, where the weight of a peak position is given by its intensity (see Section C of the Supporting Information for additional details).

**Figure 3:**
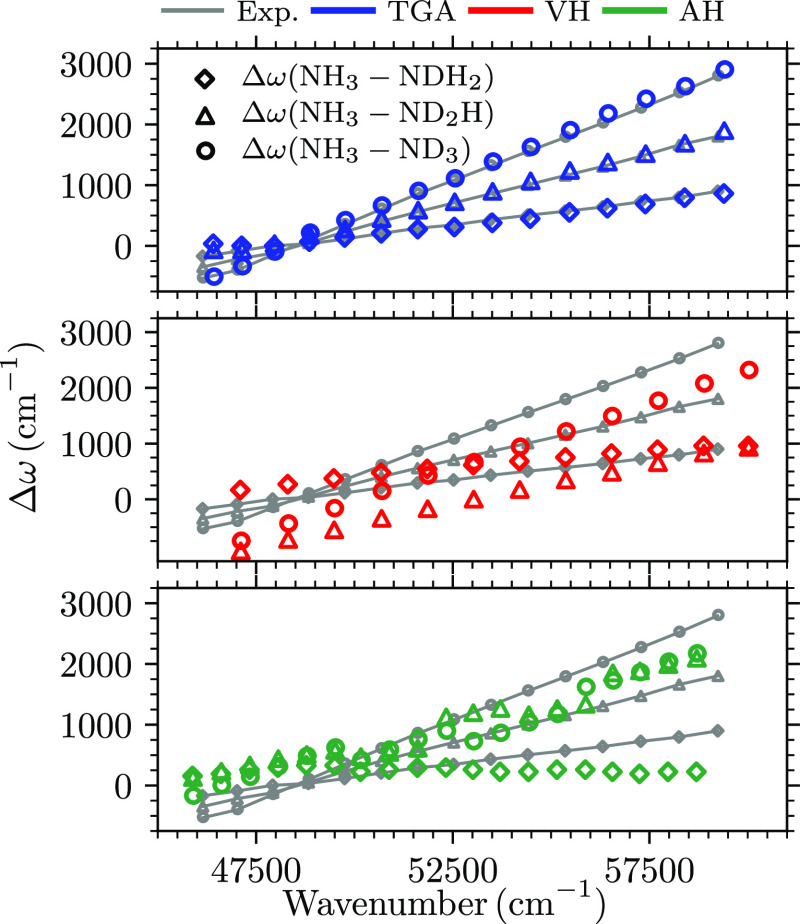
Isotope effects on the peak spacing in the  band computed with the on-the-fly TGA, VH model, and AH model are compared with the experimental values. Δω is the difference between the wavenumbers of corresponding peaks in the spectra of two isotopologues.

Within the thawed Gaussian approximation, the classical trajectory guiding the center of the wave packet follows the exact and fully anharmonic ab initio PES, whereas the width of the wave packet feels anharmonicity only approximately through the effective, locally harmonic potential. This on-the-fly approach improves over both global harmonic models and describes well the isotope effects on the peak spacing and width of the spectral envelope. The intensities of the peaks are reproduced very well near the 0–0 transition, whereas the differences are more pronounced at higher energies for all isotopologues. The isotope effect on the peak spacing in Figure 3 shows remarkable agreement with the experiment, with slight differences only near the 0–0 transition. In contrast, both harmonic models deviate substantially from the linear dependence of the shift on the peak frequency.

Despite its simplicity, the TGA predicts the peak positions accurately, exhibiting a mean absolute error (in cm^–1^) of 81 for NH_3_, 87 for NH_2_D, 63 for NHD_2_, and 53 for ND_3_. Table S5 of the Supporting Information compares the experimental and semiclassical frequencies of all peaks. Note that these frequencies exclude vertical excitation energy errors as they are computed after applying the optimal shifts of ∼200 cm^–1^ to the spectra. The better performance of the on-the-fly TGA suggests that the fully anharmonic classical trajectory employed in the TGA describes better the true periodicity of the oscillations in the quasi-bound region of the  state. Although a new calculation must be performed for each isotopologue, the results clearly indicate that this pays off.

**Figure 4:**
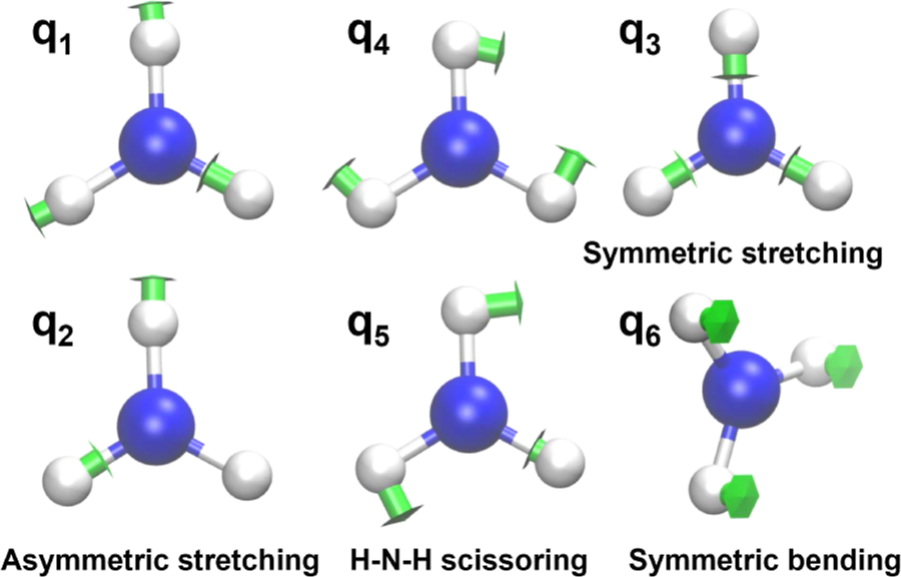
Vibrational normal modes of the excited electronic state of ammonia. In NH_3_ and ND_3_, the two asymmetric stretching modes (*q*_1_ and *q*_2_) are degenerate and the two scissoring modes (*q*_4_ and *q*_5_) are degenerate. In partially deuterated isotopologues, all normal modes are nondegenerate.

One of the strengths of semiclassical methods is the ease with which they reveal the molecular dynamics that generate these spectra. The thawed Gaussian approximation makes this interpretation even simpler because it relies on only one trajectory. Having performed all the calculations in the excited-state normal-mode coordinates (depicted in Figure 4), we also know the time evolution of each of these modes. In ammonia, the majority of the excited-state normal modes are similar to the more commonly used ground-state normal modes. In the case of NH_3_ and ND_3_, where the excited-state equilibrium geometry belongs to the *D*_3*h*_ point group, there are two pairs of degenerate normal modes—a degenerate pair of asymmetric stretching modes and a degenerate pair of scissoring modes. The other two modes—symmetric stretching and symmetric bending (umbrella motion)—are nondegenerate. In the case of partially deuterated isotopologues (NDH_2_ and ND_2_H), where the symmetry of the excited-state equilibrium geometry belongs to the *C*_2*v*_ point group, all six normal modes are nondegenerate.

**Figure 5:**
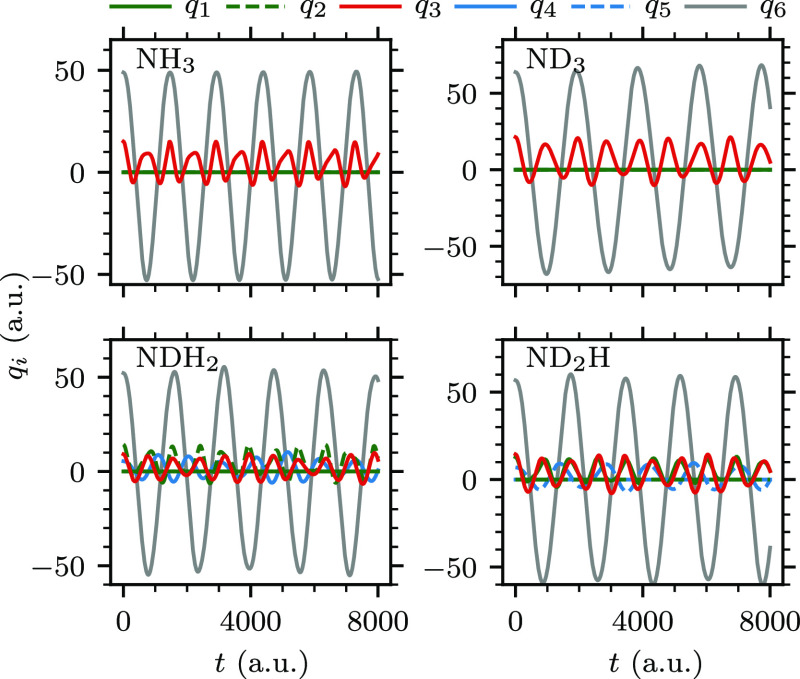
Evolution of the six excited-state normal modes in the four isotopologues of ammonia following electronic excitation at *t* = 0. The normal-mode coordinates *q*_1_ and *q*_2_ correspond to the two asymmetric stretching modes, *q*_3_ to the symmetric stretch, *q*_4_ and *q*_5_ to the two scissoring modes, and *q*_6_ to the symmetric bending (i.e., the umbrella vibration) about the minimum of the excited-state PES (see also Figure 4).

In the following, we only discuss the normal-mode evolution for the TGA, which was the only one among the considered methods that reproduced the experimental spectra accurately. Figure 5 shows the evolution of all vibrational normal modes during the propagation. As NH_3_ and ND_3_ possess the same symmetry (*D*_3*h*_), the same normal modes—symmetric stretching (*q*_3_) and symmetric bending (*q*_6_)—are activated. The symmetric stretching evolves with approximately twice the frequency of the symmetric bending mode not only in the NH_3_ and ND_3_ but also in the partially deuterated isotopologues, suggesting that the two modes are strongly coupled. Although the symmetric stretching is always excited in all isotopologues, it appears to be absent in the spectra, which has been further confirmed by jet-cooled experiments^60,61^ with NH_3_ and ND_3_. The single progression in the spectra can be explained partially by the simple fact that the bending mode is excited considerably more than other modes and partially by invoking the missing mode effect (MIME).^2,62,63^ In the MIME, the two displaced modes collude at a time *t*_M_ (with frequency ω_M_ = 2π/*t*_M_), which here happens to correspond to the progression of the symmetric bending mode. As there are more modes activated in partially deuterated isotopologues, Table 3 shows a comparison between the observed experimental frequency and the calculated MIME frequency, which indicates that in all cases the MIME is present and its frequency corresponds to the symmetric bending mode. The additionally excited vibrational normal modes in partially deuterated isotopologues are one of the asymmetric stretching modes (*q*_1_ or *q*_2_ in Figure 5) and one scissoring normal mode (*q*_4_ or *q*_5_ in Figure 5). Interestingly, one of the asymmetric stretching modes also evolves with approximately twice the frequency of the bending mode, whereas the scissoring modes are incommensurate with the rest of the modes.

**Table 3:** Mean Peak Spacing in the Experimental Spectra Compared to the MIME Wavenumber

	NH_3_	NDH_2_	ND_2_H	ND_3_
mean peak spacing	935	868	781	704
MIME wavenumber^a^	928	841	796	727

a Calculated as , where  is the nearest integer to the indicated ratio, ω_*j*_ is the wavenumber of the mode, and δ*q*_*j*_ is the displacement in the mass-scaled normal-mode coordinates (see also eq 19.2 in ref (2)). All values shown are in cm^–1^.

## Conclusions

To conclude, we have shown that even the rather simple on-the-fly ab initio thawed Gaussian approximation can capture the key isotope effects in the spectra of ammonia isotopologues. In contrast, the popular global harmonic models can reproduce some of the isotope effects, but inconsistently. The VH model, where the PES is computed in the Franck–Condon region, correctly describes the change in the width of the spectral envelope but misses the isotope effect on the peak spacing. The AH model shows two progressions instead of the single progression observed in experimental spectra. Inspection of the time evolution of excited-state normal modes shows that the single progression in the spectra can be explained by the larger excitation of the symmetric bending mode than of the other modes and by the MIME.^2,62^ We also show that, due to the change of symmetry, in partially deuterated isotopologues, additional modes are activated, even though the symmetric bending mode still dominates the dynamics and spectra.

In ammonia, the Condon approximation is sufficient. In molecules where non-Condon effects are important, they can be included with the “extended” TGA^20,32,48,64^ which propagates a Gaussian multiplied by a polynomial at zero additional cost compared to the cost of the ab initio TGA. To further improve the accuracy of semiclassical calculations of isotope effects on spectra, one should use a more sophisticated electronic structure method and one of the more accurate, multi-trajectory semiclassical methods, which can capture wave packet splitting and sometimes even tunneling. While this may be essential for more complicated systems, we have found that the ab initio thawed Gaussian approximation is sufficient for a semi-quantitative description of the isotope effects in ammonia.

## Supporting Information Available

Details of 1D harmonic model calculations, optimized geometries, harmonic frequencies, frequency shifts applied to computed spectra, normal-mode evolution for global harmonic models, comparison between on-the-fly TGA and global harmonic model autocorrelation functions, and equation for the calculation of the autocorrelation function in Hagedorn’s parametrization (PDF)
