# Supplementary material for: Isotope Effects on the Electronic Spectra of Ammonia from Ab Initio Semiclassical Dynamics
Source: J Phys Chem A. 2023 Sep 22;127(39):8117–25. doi: 10.1021/acs.jpca.3c04607 (PMC10561269; doi:10.1021/acs.jpca.3c04607)
Supplement: Supplementary file 1 — jp3c04607_si_001.pdf [file jp3c04607_si_001.pdf]

Supporting Information: Isotope Effects on the Electronic Spectra of Ammonia from Ab Initio Semiclassical Dynamics

Ēriks Klētnieks, Yannick Calvino Alonso, and Jiří J. L. Vaníček*

Laboratory of Theoretical Physical Chemistry, Institut des Sciences et Ingénierie Chimiques, Ecole Polytechnique Fédérale de Lausanne (EPFL), CH-1015, Lausanne, Switzerland

A. Computational details for 1-dimensional harmonic models

The displaced harmonic oscillator model requires constructing two potential energy surfaces (PESs). The ground- and excited-state PESs were chosen to have a vibrational frequency of 1 a.u. for the reduced mass $\mu = 1$. The excited-state surface was displaced horizontally by $\Delta q = 1$ a.u. and the vertical excitation energy was set to $\Delta E = 1.0$ a.u. In the case of the distorted harmonic oscillator model, the ground state was the same, whereas the excited state had a vibrational frequency of 2 a.u. for the reduced mass $\mu = 1$, and the horizontal and vertical displacement stayed the same.

For all the time-dependent calculations the initial wavepacket was taken to be the ground vibrational eigenstate of the ground-state PES. The total propagation time was 1000 a.u. with $\Delta t = 0.1$ a.u. The spectra were broadened with a Gaussian with a half width at half maximum of 0.05 a.u.

B. Equilibrium geometries and vibrational normal-mode frequencies

Table S1: Ground-state equilibrium geometry in Å.

	x	y	z
N	0.000	0.068	0.000
H	0.939	-0.316	0.0007
H	-0.470	-0.316	0.813
H	-0.470	-0.316	-0.813

Table S2: Ground-state vibrational normal-mode frequencies in cm^{-1}.

q_i	NH_3	NDH_2	ND_2H	ND_3
1	3594	3594	3553	2643
2	3594	3510	2643	2642
3	3461	2582	2525	2471
4	1667	1635	1492	1210
5	1667	1420	1264	1210
6	1048	967	876	796

Table S3: Excited-state equilibrium geometry in Å.

	x	y	z
N	0.000	0.000	0.000
H	1.046	0.000	0.000
H	-0.523	0.000	0.906
H	-0.523	0.000	-0.906

Table S4: Excited-state vibrational normal mode frequencies in cm^{-1}.

q_i	NH_3	NDH_2	ND_2H	ND_3
1	3006	3005	2952	2237
2	3006	2891	2240	2237
3	2812	2145	2061	1988
4	1344	1334	1225	980
5	1344	1122	988	980
6	732	680	623	561

C. Details of the calculations of the spectra and isotope effects

Table S5: Peak energies in cm^{-1} acquired from the on-the-fly calculation after the application of the optimal shift. The peak energies in bold indicate the peak that was used to obtain the best fit. The mean absolute error between the experiment and the calculation is provided at the bottom of the Table together with the root mean square error (RMSE).

	NH_3		NH_2D		NHD_2		ND_3	
Peak number	Exp.	Calc.	Exp.	Calc.	Exp.	Calc.	Exp.	Calc.
1	46,134	46,403	46,305	46,371	46,481	46,468	46,666	46,929
2	47,010	47,127	47,101	47,129	47,228	47,192	47,411	47,480
3	47,916	47,953	47,913	47,955	48,035	47,916	48,068	48,065
4	48,823	48,849	48,790	48,782	48,776	48,673	48,728	48,651
5	49,746	49,744	49,633	49,608	49,515	49,465	49,397	49,339
6	**50,674**	50,674	**50,469**	50,469	50,271	50,223	50,073	50,028
7	51,594	51,604	51,303	51,330	51,036	51,015	50,741	50,717
8	52,515	52,534	52,170	52,226	**51,808**	51,808	**51,438**	51,438
9	53,459	53,498	53,028	53,121	52,601	52,599	52,143	52,129
10	54,401	54,462	53,897	54,017	53,396	53,392	52,849	52,852
11	55,353	55,461	54,783	54,912	54,195	54,218	53,568	53,576
12	56,306	56,426	55,667	55,808	54,999	55,045	54,286	54,264
13	57,274	57,390	56,548	56,703	55,800	55,871	55,009	54,988
14	58,248	58,389	57,451	57,599	56,587	56,698	55,729	55,780
15	59,235	59,387	58,336	58,528	57,432	57,490	56,440	56,503
16			59,263	59,424	58,146	58,282	57,162	57,226
17					58,983	59,212	57,884	57,950
18							58,610	58,673
19							59,340	59,431
Mean abs. error	81		87		63		53	
RMSE	74		63		60		58	

Table S6: Energy shifts applied to all the spectra in cm^{-1}.

	NH_3	NDH_2	ND_2H	ND_3
Adiabatic harmonic	-230	-494	-596	-418
Vertical harmonic	-151	-390	403	151
On-the-fly *ab initio*	-496	-397	-355	-242

The width of the spectral envelope σ, presented in the main text, was estimated as twice the square root of the weighted variance of the peak positions

$$\sigma = 2\sqrt{\mathrm{Var}(x)}, \tag{1}$$

$$\mathrm{Var}(x) = \frac{1}{n}\sum_{i=1}^{n}(x_i - \bar{x})^2, \tag{2}$$

$$\bar{x} = \frac{\sum_{i=1}^{n} w_i x_i}{\sum_{i=1}^{n} w_i}, \tag{3}$$

where the weight of a peak position w_i is given by its intensity and x_i is the peak position.

D. Normal mode evolution and autocorrelation function in harmonic models

The normal mode evolution for the adiabatic and vertical models is displayed in Fig. S1 and S2. The normal modes shown here are defined by the mass-scaled excited-state Hessian. The corresponding wavepacket autocorrelation functions are compared in Fig. S3

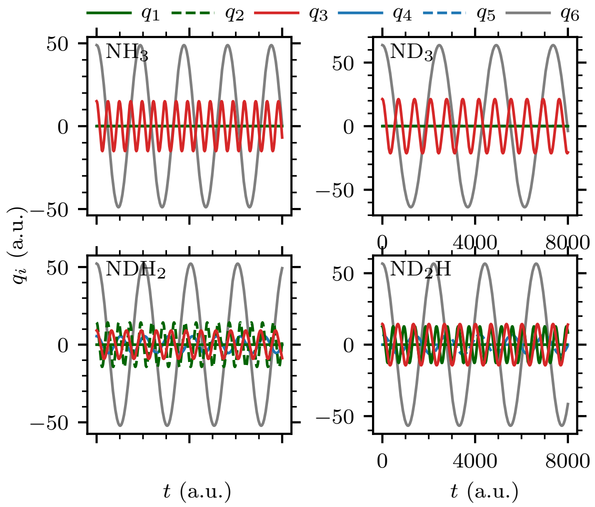

Figure S1: Evolution of excited-state normal modes in the adiabatic harmonic model.

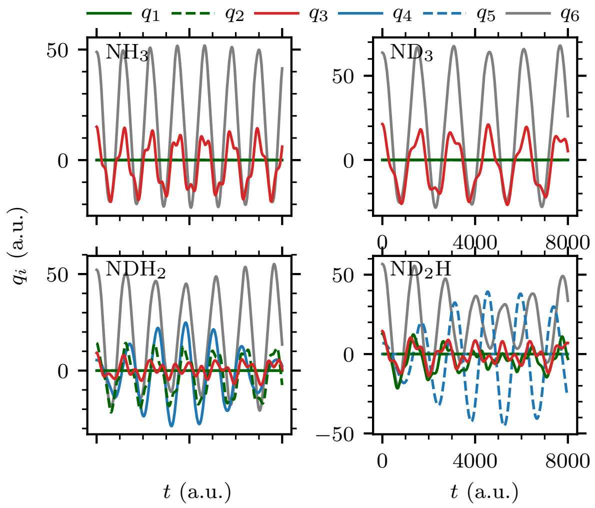

Figure S2: Evolution of excited-state normal modes in the vertical harmonic model.

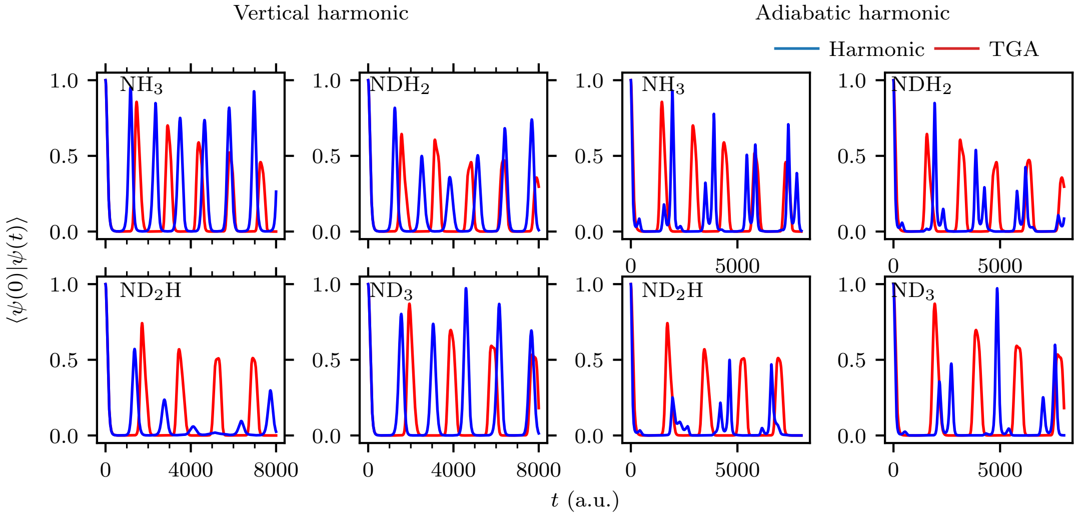

Figure S3: Wavepacket autocorrelation functions of on-the-fly TGA and global harmonic models as a function of total propagation time.

E. Computation of the wavepacket autocorrelation function

The wavepacket autocorrelation function $C(t) = \langle\psi(0)|\psi(t)\rangle$ was calculated at each time step using the analytical expression

$$\langle\psi_1|\psi_2\rangle = \sqrt{\frac{1}{-\frac{i}{2}\det[P_2 \cdot Q_1^* - P_1^* \cdot Q_2]}} \times \exp\Big\{\frac{1}{\hbar}\Big[-\frac{1}{2}\delta\xi^T \cdot \delta A^{-1} \cdot \delta\xi + \delta\eta\Big]\Big\} \tag{4}$$

for the overlap of arbitrary two Gaussians in Hagedorn representation.

In Eq. 4 $\delta\lambda := \lambda_2 - \lambda_1^*$ for $\lambda_j = A, \xi, \eta$ and

$$\xi_j = p_j - P_j \cdot Q_j^{-1} \cdot q_j, \tag{5}$$

$$A_j = P_j \cdot Q_j^{-1}, \tag{6}$$

$$\eta_j = \frac{1}{2} q_j^T \cdot P_{2=j} \cdot Q_j^{-1} \cdot q_j - q_j^T \cdot p_j + S_j. \tag{7}$$

State ψ_1 corresponds to the initial wavepacket at time 0, and ψ_2 is the wavepacket that has been evolved for time t.
